# Ultrasonography in the assessment of primary myxofibrosarcoma in the left atrium: a case report

**DOI:** 10.1186/s12872-022-03009-6

**Published:** 2022-12-19

**Authors:** Xiaoguang Huo, Wei Zhao, Xiao Liu, Wenzhong Zhang, Li Xu, Baohua Zhang, Ju Chen

**Affiliations:** 1 Department of Ultrasound, Zibo Central Hospital, No. 54 West Communist Youth League Road, Zhangdian District, Zibo, 255036 Shandong People’s Republic of China; 2 Department of Pathology, Zibo Central Hospital, No. 54 West Communist Youth League Road, Zhangdian District, Zibo, 255036 Shandong People’s Republic of China

**Keywords:** Myxofibrosarcoma, Left atrium, Ultrasound

## Abstract

**Background:**

Cardiac myxofibrosarcoma is a rare cardiac malignant tumor, whose diagnosis is challenging due to its rare and non-specific manifestations. Ultrasound is the most important tool for detecting cardiac tumors. Yet, its diagnostic value in cardiac myxoidfibrosarcoma is rarely reported. Herein, we summarized the ultrasonic manifestations of myxofibrosarcoma in a 72-year-old Han woman.

**Case presentation:**

The patient presented with crushing chest pain without obvious inducement, lasting 3–5 min each time, which would be relieved after rest, accompanied by palpitation, chest tightness, shortness of breath, dizziness, and syncope. The electrocardiogram (ECG) suggested atrial fibrillation. Ultrasound found two moderate echogenic masses in the left atrium; one was about 48 × 31 mm in size, and the other was about 25 × 24 mm in size. The clinical diagnosis was atrial mass and atrial fibrillation. The patient underwent the operation of left atrial tumor resection + mitral valvuloplasty + atrial fibrillation radiofrequency ablation + left atrial appendectomy. The tumor was completely removed, and the patient did not receive radiotherapy or chemotherapy after surgery. The patient was reexamined by ultrasound at 6, 42, and 91 days after surgery, and no obvious abnormalities were found. On day 115, moderate echoic mass was detected on the posterior wall of the left atrium, nearing the mitral valve ring, with a size of about 28 × 23 mm. Currently, the patient is under follow-up care.

**Conclusion:**

As the most important examination method for cardiac tumors, cardiac ultrasound has good diagnostic and differential diagnosis value and can be used regularly due to its simplicity and safety. The diagnosis rate of cardiac myxofibrosarcoma can be greatly improved by summarizing the ultrasonographic manifestations of cardiac myxofibrosarcoma and differentiating them from other lesions.

## Background

Primary cardiac tumors are relatively rare, with a prevalence of only about 0.002–0.03%, where benign tumors account for about 75% of all cases and malignant tumors account for about 25% [1]. Moreover, 95% of malignancies are sarcomas, with angiosarcoma being the most common type [2]. Myxofibrosarcoma rarely occurs in the heart. Primary cardiac myxofibrosarcoma is very aggressive. So far, only a few cases have been reported [3–5]; these studies reported clinical manifestations, pathological features, and treatment options. The diagnostic value of ultrasound has been rarely reported. Herein, we summarized the ultrasonic manifestations of myxofibrosarcoma in single case in conjunction with previous relevant literature, aiming to provide a theoretical basis for the diagnosis and treatment of this type of tumor.

## Case presentation

**Fig. 1:**
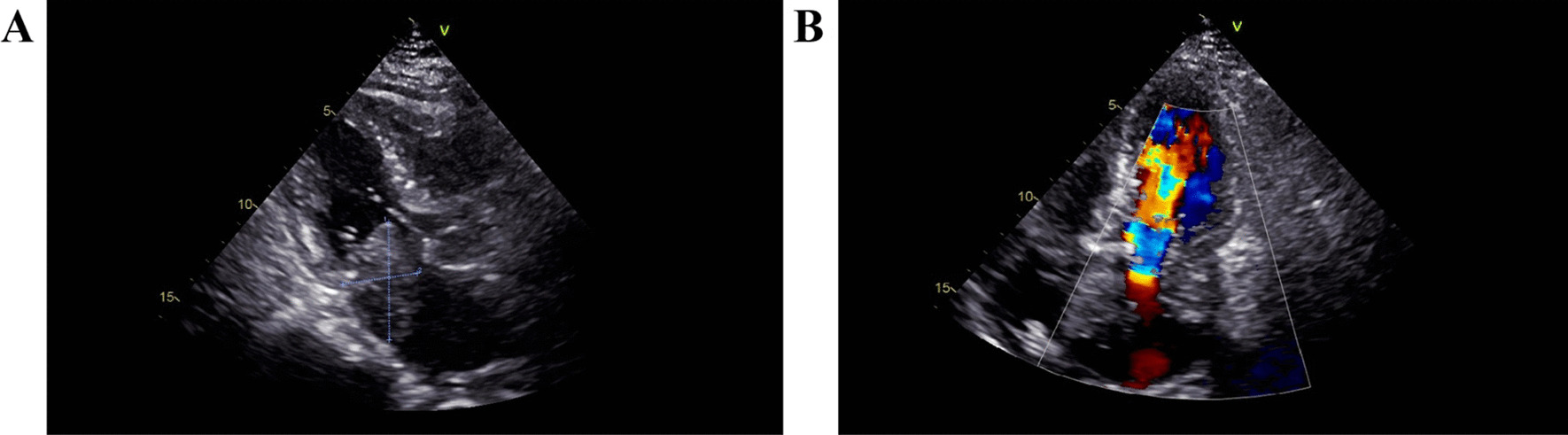
Preoperative ultrasound image of myxofibrosarcoma in the left atrium. (**A**) Two-dimensional ultrasound image of myxofibrosarcoma. (**B**) Ultrasound image of diastolic mitral flow in myxofibrosarcoma

The patient was a 72-year-old woman who presented with chest pain without obvious inducement (lasting 3–5 min each time and would relieve following rest) accompanied by palpitation, chest tightness, shortness of breath, dizziness, and syncope. The patient had a history of diabetes for more than 10 years, and she regularly took metformin. Her blood sugar was within the normal range. The patient denied a family history of heart cancer. There were no obvious abnormalities on a cardiac ultrasound examination that was performed one year ago. Auscultation of the heart showed that the pulse was deficient, the heart rhythm was absolutely irregular, and the heart sound was erratic, indicating atrial fibrillation. There was no pericardial fricative rub. The N-terminal B-type natriuretic peptide precursor was 1010.0 pg/ml (normal range 0–125 pg/ml). The electrocardiogram (ECG) suggested atrial fibrillation. An emergency ultrasound indicated an enlarged left atrium. The anteroposterior diameter of the left room was about 42 mm. Two moderate echogenic masses were found in the left atrium; one was about 48 × 31 mm in size and was closely related to the posterior wall of the left atrium and the mitral lobe ring, and the other was about 25 × 24 mm in size and was attached to the middle of the atrial septum. The masses had irregular morphology, clear boundary, poorly uniform echo, small deformation, and a wide base. The larger mass caused a mild obstruction of the inflow tract during the diastolic phase (Fig. 1). The left ventricular ejection fraction was about 60%. Ultrasound was more likely to consider tumors. The clinical diagnosis was an atrial tumor and atrial fibrillation.

The patient underwent left atrial tumor resection + mitral valvuloplasty + atrial fibrillation radiofrequency ablation + left atrial appendectomy on December 3, 2021. During the operation, a huge tumor was located in the left atrium, about 8 × 5 cm. The tumor was partially solid and partly jelly-like; it invaded the junction of the left upper pulmonary vein and the left lower pulmonary vein, the left atrial base, the atrial septum, and the mitral valve ring. The tumor and part of the lesion, atrial septum, and mitral valve ring were removed by surgery. The patient did not receive radiotherapy or chemotherapy after surgery.

**Fig. 2:**
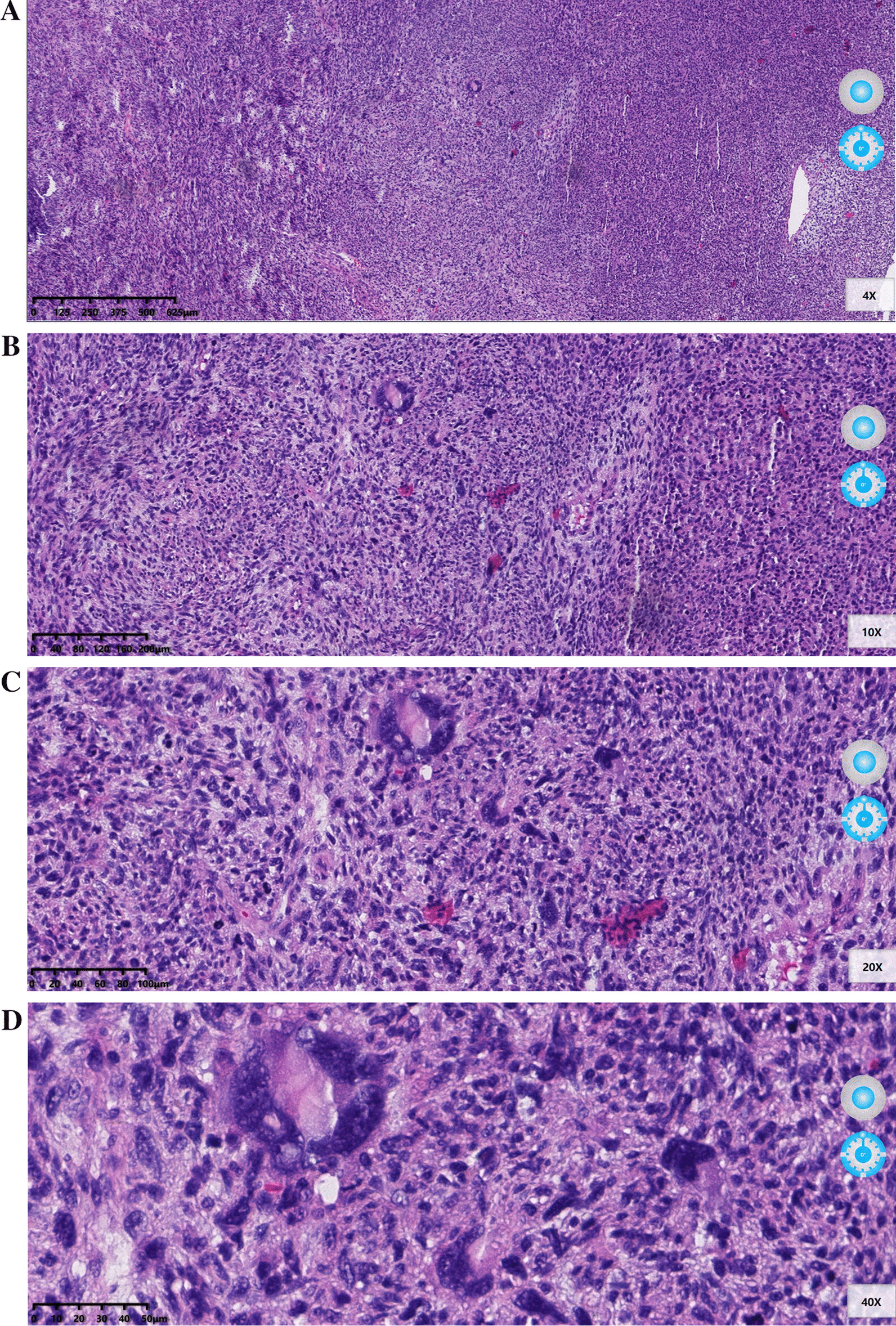
The pathologic image of myxofibrosarcoma. (**A**) Low power image: Spindle tumor cells are arranged in interwoven bundles, with large density and compact arrangement (haematoxylin–eosin, original magnification: 4 ×). (**B**–**D**) High power image: the nucleus of the tumor is large, hyperchromatic, and heteromorphic. Mitosis is more common, and giant tumor cells are seen (haematoxylin–eosin, original magnification: 10 × , 20 × , 40 ×)

Pathological findings were as follows: there were 4 masses in the left atrium, the largest of which was about 5 × 5 × 2.5 cm. The mass section was grayish-red, some of which were gelatinous and slightly tough. The tumor was diagnosed as a malignant tumor derived from spindle cells of soft tissue. Combined with immunohistochemistry, myxofibrosarcoma with partial differentiation into leiomyosarcoma and multifocal hemorrhage and necrosis were considered (Fig. 2). Vimentin, Bcl-2, CD99, CDK4, MDM2, SMA, Desmin, STAT6 and Ki-67 were positive, while CD31, CD34, D2-40, S-100, EMA, and dog-1 were negative. Pathological images were obtained with a digital sectioning scanner KF-PRO-005 (the digital slice reading software K-ViEWER was used and the image resolution was 120Dpi).

**Fig. 3:**
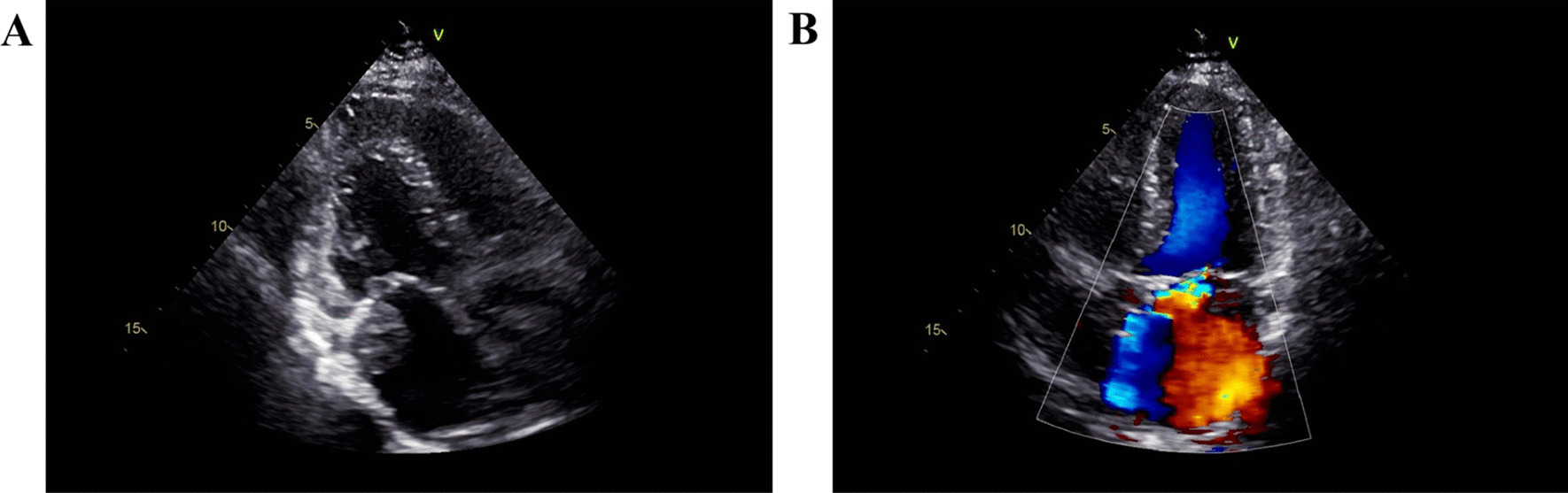
Postoperative ultrasound image of myxofibrosarcoma in the left atrium. (**A**) Two-dimensional ultrasound image of myxofibrosarcoma. (**B**) Ultrasound image of diastolic mitral flow in myxofibrosarcoma

The patients were reexamined by ultrasound at 6, 42, and 91 days after surgery, and no obvious abnormalities were found. On day 115 post-surgery, a moderate echoic mass was detected on the posterior wall of the left atrium, nearing the mitral valve ring, with a size of about 28 × 23 mm. The mass was well-bounded, irregular in shape, uneven in echo, and broad in base. Ultrasound suggested that the mass in the left atrium was a tumor, as shown in Fig. 3. The patient showed no symptoms, such as chest tightness. Due to the patient's age, the family refused further treatment. The last follow-up time was 6 months after surgery. The patient is still alive and is currently being followed up.

## Discussion and conclusions

Myxofibrosarcoma was first described in 1977 by Angervall et al*.* [6]. It is a rare soft tissue malignancy with a high recurrence rate that usually occurs in elderly men, mostly in the limbs, and rarely in the heart. It may affect any of the four heart chambers but is more common in the left atrium [7]. The clinical manifestation of cardiac myxofibrosarcoma depends on the size and anatomic location of the tumor. The main manifestations are hemodynamic abnormalities, including heart murmurs, cardiac insufficiency, syncope or sudden death, and embolism caused by the shedding of the tumor itself and its fragments [8]. The clinical presentation of left atrial myxofibrosarcoma may resemble left atrial myxoma. Myxofibrosarcoma of the left atrium can cause mitral valve obstruction and cause symptoms of mitral stenoses, such as dyspnea, cough, hemoptysis, and so on [9]. In this case, chest pain and tightness were the main symptoms.

Cardiac myxofibrosarcoma is a rare cardiac malignancy with rapid progression, so early detection and treatment are of crucial importance. Cardiac ultrasound examination can identify the tumor's location, scope, size, shape, and the relationship with the surrounding tissue and dynamically assess the activity pattern of tumors and the degree of dysfunction caused by it. It is an indispensable diagnostic technology for identifying the types of cardiac tumor. Cardiac ultrasound is divided into transthoracic ultrasound and transesophageal ultrasound. Transesophageal ultrasound is not affected by external factors, such as obesity and thoracic deformity. It also has a higher resolution of the tissue structure and can obtain more anatomical information about the heart. Unfortunately, our patient could not tolerate transesophageal ultrasound due to age and physical reasons.

The main ultrasonic manifestations of myxofibrosarcoma in the left atrium are low or moderate echogenicity mass attached to the valve or atrium wall in the left atrium, with unclear boundary with attachment, irregular shape, and uneven echogenicity. In addition, the tumor is generally large, with no pedicle and a wide base, which can invade pulmonary veins, etc. [10, 11]. Some patients are complicated with pericardial effusion. Differential diagnosis is required with the following diseases: (1) left atrial myxoma: most myxomas are attached to the edge of the oval fossa, and very few are attached to the atrial wall, valves, etc. The tumor body is mostly oval or round; the echo is relatively uniform, and the pedicle is relatively narrow and clear. Myxoma has a high degree of activity and can move with diastolic contractions [12, 13]. (2) Left atrial thrombus: thrombus is often accompanied by the primary disease. Most thrombi occur in the left atrial posterior wall, lateral wall, and left atrial appendage; a few can extend to the atrial septum. A thrombus is generally oval or irregular in shape. Its echo behaves differently over time. The thrombus is sessile, and its base is usually broad. Old thrombus does not move with diastolic cardiac contraction. However, fresh thrombus can slightly change, and can also fall off and free in the left atrium. In addition, the phenomenon of spontaneous development can be observed in the heart cavity. It can be greatly changed after anticoagulant application [14]. (3) Atrioventricular valve vegetation often occurs in patients with a history of primary diseases, such as rheumatic heart disease and infective endocarditis. Vegetation mostly occurs in the part of blood flow impact or local vortex, such as the atrial surface with incomplete mitral valve closure, the ventricular surface with incomplete aortic valve closure, etc. Vegetation varies in size but is usually small and varied in shape, and its echo varies with time. Discontinuous areas of echo can be found in newly formed vegetations, suggesting abscess formation. Complications, such as valve perforation, pseudovalvular tumor, fistula, valve regurgitation, and similar, can also occur [15, 16].

Magnetic resonance imaging (MRI) has been reported to be useful for classifying myxofibrosarcoma. Mucous components are predominant in low-grade myxofibrosarcoma while less in middle and high-grade myxofibrosarcoma. T1-weighted images of mucus components showed a slightly lower signal number, while their T2-weighted images showed a high signal and mild enhancement after enhancement. In some cases, serrated, meshlike, and mild patchy enhancement was observed [17]. Unlike myxoid degeneration, cystic degeneration and necrotic areas are more common in highly malignant myxofibrosarcoma without enhancement in enhanced scanning [18]. Lefkowitz et al*.* [19] suggested that the "tail sign" of myxofibrosarcoma was related to the malignancy of the tumor. The growth pattern of myxofibrosarcoma along fascia, neurovascular, or muscle tissue resulted in abnormal signals in MR T2-weighted images, enhanced MRI, and short-time inversion recovery (STIR) sequence images. It manifested as a curvilinear signal with a clear boundary and gradually tapering, namely, "tail sign", which was the most common imaging manifestation of myxofibrosarcoma, with a reported occurrence rate of 64–77% [19, 20]. To sum up, MRI figures have prominent importance in the grading of myxofibrosarcoma, which cannot be matched by ultrasound. Unfortunately, in this case, the patient was claustrophobic and unsubtible to undergo an MRI examination.

Fludeoxyglucose (FDG)-positron emission tomography (PET)/computed tomography (CT) is another useful non-invasive imaged approach that can be used to accurately detect the number, location, and distant metastasis of primary cardiac tumors. FDG PET/CT imaging evaluates the glucose metabolism status of tumor tissues at the molecular level, reflects cell activity, and provides functional metabolism information beyond anatomical imaging [21]. FDG PET/CT imaging can provide information regarding tumor staging, guide tumor treatment, and evaluate its efficacy. However, due to its high expenditure, FDG PET/CT imaging is not considered as a routine test. In this case, the patient refused to undergo this test. Although MRI and PET/CT have unique advantages in diagnosing myxofibrosarcoma, these two tests cannot be used as routine examinations, mainly due to their high cost, and long time. On this basis, ultrasound should be considered as the first choice for myxofibrosarcoma imaging examination.

Although ultrasound, MRI, and PET/CT can provide an important basis for diagnosing myxofibrosarcoma, the final diagnosis depends on pathology. According to relevant literature reports, vimentin, CD34, and Ki-67 are mostly positive or strongly positive in myxofibrosarcoma, among which vimentin is the most characteristic for diagnosing high-grade myxofibrosarcoma [22]. In this case, vimentin and Ki-67 were positive and CD34 was negative. Cardiac myxofibrosarcoma progresses rapidly and has serious consequences due to its pathological characteristics. Therefore, surgery is still the main treatment for primary left atrial malignant tumor [23]. The effectiveness of adjuvant chemotherapy and radiotherapy is still controversial [24]. Therefore, early and complete surgical resection offers the best chance of survival.

Cardiac myxofibrosarcoma is a rare cardiac malignancy whose diagnosis is challenging. Cardiac ultrasound has a good diagnostic and differential diagnosis; it is simple and safe to perform; the diagnosis rate of cardiac myxofibrosarcoma can be greatly improved by summarizing the ultrasonographic manifestations of cardiac myxofibrosarcoma and differentiating it from other lesions. The combination of ultrasound, clinical manifestations, and pathological findings can provide a valuable reference for diagnosing and treating myxofibrosarcoma.

## Data Availability

The data supporting this study's findings are available from the corresponding author on request.

## Abbreviations

ECG: Electrocardiogram
MRI: Magnetic resonance imaging
STIR: Short-time inversion recovery
FDG: Fludeoxyglucose
PET: Positron emission tomography
CT: Computed tomography
